# Dynamic changes of microbial flora and therapeutic consequences in persistent peritonitis

**DOI:** 10.1186/s13054-015-0789-9

**Published:** 2015-03-02

**Authors:** Philippe Montravers, Guillaume Dufour, Jean Guglielminotti, Mathieu Desmard, Claudette Muller, Hamda Houissa, Nicolas Allou, Jean-Pierre Marmuse, Pascal Augustin

**Affiliations:** Département d’Anesthésie Réanimation, Université Paris Diderot, APHP, CHU Bichat-Claude Bernard, 46, Rue Henri Huchard, Paris, 75018 France; Université Paris Diderot, APHP, CHU Bichat-Claude Bernard, Laboratoire de Microbiologie, Paris, France; Université Paris Diderot, APHP, CHU Bichat-Claude Bernard, Service de Chirurgie Générale, Paris, France

## Abstract

**Introduction:**

Persistent peritonitis is a frequent complication of secondary peritonitis requiring additional reoperations and antibiotic therapy. This situation raises specific concerns due to microbiological changes in peritoneal samples, especially the emergence of multidrug-resistant (MDR) strains. Although this complication has been extensively studied, the rate and dynamics of MDR strains have rarely been analysed.

**Methods:**

We compared the clinical, microbiological and therapeutic data of consecutive ICU patients admitted for postoperative peritonitis either without subsequent reoperation (n = 122) or who underwent repeated surgery for persistent peritonitis with positive peritoneal fluid cultures (n = 98). Data collected on index surgery for the treatment of postoperative peritonitis were compared between these two groups. In the patients with persistent peritonitis, the data obtained at the first, second and third reoperations were compared with those of index surgery. Risk factors for emergence of MDR strains were assessed.

**Results:**

At the time of index surgery, no parameters were able to differentiate patients with or without persistent peritonitis except for increased severity and high proportions of fungal isolates in the persistent peritonitis group. The mean time to reoperation was similar from the first to the third reoperation (range: 5 to 6 days). Septic shock was the main clinical expression of persistent peritonitis. A progressive shift of peritoneal flora was observed with the number of reoperations, comprising extinction of susceptible strains and emergence of 85 MDR strains. The proportion of patients harbouring MDR strains increased from 41% at index surgery, to 49% at the first, 54% at the second (*P* = 0.037) and 76% at the third reoperation (*P* = 0.003 versus index surgery). In multivariate analysis, the only risk factor for emergence of MDR strains was time to reoperation (OR 1.19 per day, 95%CI (1.08 to 1.33), *P* = 0.0006).

**Conclusions:**

Initial severity, presence of Candida in surgical samples and inadequate source control are the major risk factors for persistent peritonitis. Emergence of MDR bacteria is frequent and increases progressively with the number of reoperations. No link was demonstrated between emergence of MDR strains and antibiotic regimens, while source control and its timing appeared to be major determinants of emergence of MDR strains.

## Introduction

Persistent peritonitis is a dreaded complication reported in 20 to 58% of all cases of secondary peritonitis [1–8]. The diagnosis is suspected in the absence of clinical improvement or in the presence of clinical worsening ≥24 hours after presumed adequate surgical source control, antimicrobial and medical management and exclusion of extra-abdominal sources of sepsis [8]. This entity, also called tertiary peritonitis, requires additional reoperations, antibiotic therapy and management of organ dysfunction [9,10]. Most publications are based on limited numbers of cases, with limited microbiological data and therapeutic aspects are rarely analysed [2,3,5,7,11].

Recent reports of postoperative peritonitis have demonstrated the emergence of multidrug-resistant (MDR) bacteria [12-15]. Many of these patients develop persistent peritonitis [8] and undergo repeated surgery and prolonged anti-infective treatments. These patients represent a specific population in which the dynamic changes of microbiological samples in the peritoneal space can be more closely documented and the effects of antibiotic pressure can be assessed, especially on emerging MDR strains.

In order to clarify these issues, we compared consecutive ICU patients admitted for postoperative peritonitis who did not undergo subsequent reoperation and those who underwent repeated surgery for persistent peritonitis. Clinical and microbiological features of these patients were compared. The resistance profile of these patients with persistent peritonitis, the dynamic changes and their therapeutic features were analysed during the course of repeated surgeries, with specific focus on the risk factors for emergence of MDR strains.

## Materials and methods

### Study population

From January 1999 through December 2007, all consecutive patients admitted to our ICU for the management of postoperative peritonitis were prospectively entered into a database. The study was approved by the local institutional review board (CEERB CHU Bichat Paris VII University, APHP, Paris), which waived the need for signed informed consent.

### Selection of cases and inclusion criteria

**Figure 1:**
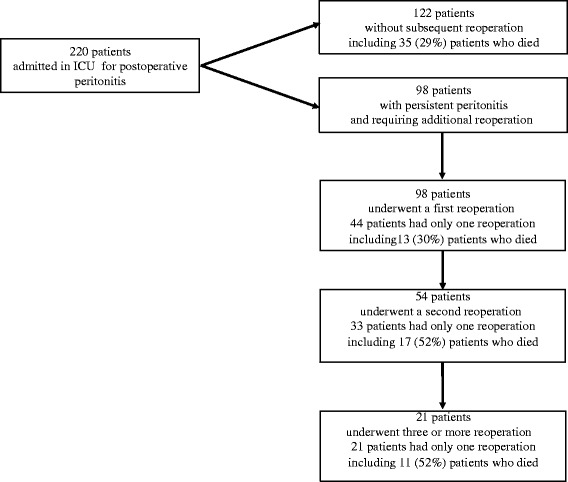
**Flow chart of the 220 patients studied.**

During the study period, 220 consecutive patients were admitted to our ICU with a diagnosis of postoperative peritonitis and underwent the index procedure for management of infection. One hundred and twenty-two patients (55%) required only the index operation, while 98 patients (45%) underwent the index procedure and additional reoperation for confirmed positive bacterial and/or fungal cultures of peritoneal samples (Figure 1) [8]. All these patients required reoperation for signs of persistent peritonitis suspected on the basis of prolonged fever, abdominal tenderness, absent bowel sounds, sustained organ dysfunction, while extra-abdominal sepsis was ruled out. The diagnosis made at reoperation was based on the discovery of purulent material distributed throughout the abdominal cavity, suture leakage, bowel perforation, purulent collections or positive bacteriological cultures of intraoperative peritoneal fluid samples. All 98 patients with persistent peritonitis underwent a first repeat laparotomy, some required a second repeat and a smaller number required a third repeat (Figure 1).

All patients with surgical intra-abdominal infection who required reoperation were included, except for patients with acute infectious pancreatitis or primary peritonitis, and transplant recipients. Patients for whom microbiological samples were not collected at the index surgery (S0) were excluded from the analysis. In our therapeutic strategy, percutaneous drainage is not considered in patients presenting signs of uncontrolled sepsis [16]. No selective decontamination of the digestive tract was performed.

### Surgical procedures

Surgical principles were those used for septic surgery. All patients underwent midline laparotomy with complete exploration of the peritoneal cavity. Gross purulent exudates were aspirated, and debris and particles were removed, followed by abundant peritoneal lavage. Ostomy was preferred to primary anastomosis. No open-wound management was performed and the abdomen was not irrigated after surgery.

### Microbiological data

Peritoneal fluid samples were systematically collected during surgery and immediately sent for microbiological examination. Cultures were performed with identification and susceptibility testing for Gram-positive and negative aerobic bacteria, anaerobes and fungi. Antibiotic susceptibility was recorded for each organism determined by the disk-diffusion method, according to the criteria of the Antibiogram Committee of the French Society for Microbiology [17]. MDR bacteria were defined as those resistant to three or more antimicrobial classes [18]: methicillin-resistant *Staphylococcus aureus* (MRSA) and coagulase-negative staphylococci (MRCNS); ampicillin-resistant enterococci; *Enterobacteriaceae* producing an extended-spectrum beta-lactamase or producing a derepressed cephalosporinase; non-fermenting Gram-negative bacilli (NFGNB) resistant to piperacillin/tazobactam, ceftazidime, or imipenem/cilastatin, or producing an extended-spectrum beta-lactamase.

Microbiological samples collected during reoperation (at the first, second and third reoperation) were compared with those obtained on the index procedure in the group of patients with persistent abdominal sepsis. Emergence was defined as the culture of a new organism (bacteria or fungi) from one sample to the next on the basis of identification and susceptibility testing. No analysis of genetic relatedness was performed.

### Antibiotic therapy

Empirical anti-infective therapy, systematically initiated at the index operation, took into account the clinical severity and usually consisted of a combination of piperacillin/tazobactam or imipenem/cilastatin with amikacin and vancomycin [12], possibly associated with antifungal therapy (mainly fluconazole) based on presumed risk factors [19]. Definitive anti-infective therapy, adapted to the results of identification and antibiotic susceptibility testing (≥48 hours), was defined as adequate when it targeted all cultured organisms. The same procedure was applied at each reoperation in patients with persistent peritonitis.

### Data collection

All patient charts were reviewed. Demographic data and severity scores (simplified acute physiology score II (SAPS II) [20] were recorded on admission to ICU. The severity of the underlying medical condition [21] (malignancy, diabetes mellitus, immunosuppression) was assessed. The characteristics of the initial intra-abdominal infection were recorded: aetiology, primary site of infection (above/below transverse mesocolon). The clinical and severity characteristics at the time of index operation and each reoperation (first to third reoperation) were assessed: temperature, white blood cell count (WBC), serum creatinine and sequential organ failure assessment (SOFA) score [22].

### Statistical analysis

Results are expressed as mean ± 1 SD or number and proportion. Statistical significance was defined as *P* <0.05. Statistical analysis was performed using R software, version 2.14.1 (R Foundation for Statistical Computing, Vienna, Austria). Comparisons between groups were made using the chi-square and Fisher’s exact test for discrete variables and analysis of variance (ANOVA) for continuous variables.

Risk factors for emerging microorganisms and for emerging MDR organisms were analysed in univariate analysis with the unpaired Wilcoxon test for continuous variables, Fisher’s exact test for discrete variables and univariate logistic regression. Odds ratios (ORs) and 95% CI were calculated. Variables with a *P*-value <0.2 in univariate analysis were entered in a multivariate logistic regression analysis with backward selection. The model was evaluated for discrimination with the *c*-statistic and for calibration with the Hosmer-Lemeshow test.

## Results

### Comparison between patients without and with persistent abdominal sepsis

The clinical characteristics of the 122 patients without reoperation and the 98 patients with persistent abdominal sepsis are compared in Table 1. At the time of the index operation, significantly increased severity and higher proportions of bowel perforation were the most relevant parameters in the patients who underwent reoperation. No specific parameter related to underlying disease, demographic characteristics, intraoperative discoveries, surgical techniques or perioperative management was able to identify patients at risk of reoperation. Ostomy was performed at the time of the index surgery in 116 cases (53%) (Table 1). We did not observe any significant link between ostomy and failure of source control.

**Table 1:** **Characteristics of the patients without and with persistent peritonitis at the time of ICU admission for initial septic surgery**

	**Without persistent peritonitis**	**Persistent peritonitis**	***P***
	**n = 122**	**n = 98**	
Male gender, n (%)	59 (48)	58 (59)	0.10
Age, mean ± SD	62 ± 18	62 ± 14	0.99
Comorbidities			
Cancer, n (%)	51 (42)	35 (36)	0.35
Diabetes, n (%)	21 (17)	10 (10)	0.17
Immunosuppression, n (%)	17 (14)	9 (9)	0.29
SAPS II score, mean ± SD	49 ± 23	50 ± 16	0.85
SOFA score, mean ± SD	6.8 ± 3.9	8.2 ± 3.2	0.01
Haemodynamic failure^1^, n (%)	49 (40)	59 (60)	0.003
Respiratory failure^1^, n (%)	32 (26)	36 (37)	0.09
Renal failure^1^, n (%)	16 (13)	22 (22)	0.07
Initial procedure leading to postoperative peritonitis			
Emergency surgery, n (%)	43 (35)	47 (48)	0.056
Elective surgery, n (%)	79 (65)	51 (52)	-
Characteristics of the initial surgical procedure			
Gastroduodenal surgery, n (%)	32 (26)	31 (32)	0.37
Colonic surgery, n (%)	48 (39)	45 (46)	0.32
Small bowel surgery, n (%)	20 (16)	19 (19)	0.59
Other type of surgery, n (%)	22 (18)	11 (11)	0.18
Aetiology of postoperative peritonitis			
Bowel perforation, n (%)	42 (34)	48 (49)	0.029
Anastomotic leakage, n (%)	42 (34)	30 (30)	0.54
Purulent collection, n (%)	17 (14)	20 (20)	0.21
Other causes, n (%)	21 (18)	19 (19)	0.72
Primary site of infection above transverse mesocolon, n (%)	30 (25)	32 (33)	0.18
Colonic site of infection, n (%)	40 (33)	35 (36)	0.65
Small bowel site of infection, n (%)	49 (41)	32 (33)	0.25
Gastric site of infection, n (%)	26 (21)	25 (26)	0.46
Creation of ostomy, n (%)	62 (53)	54 (47)	0.52
Adequacy of empirical antibiotic therapy, n (%)	79 (65)	63 (64)	0.94
Use of combination of antibiotic therapy, n (%)	88 (72)	84 (86)	0.02
Use of carbapenems, n (%)	24 (20)	29 (30)	0.09
Use of piperacillin/tazobactam, n (%)	84 (69)	62 (63)	0.38
Use of vancomycin, n (%)	43 (35)	50 (51)	0.02
Use of aminoglycosides, n (%)	50 (41)	53 (54)	0.053
Use of empirical antifungal therapy, n (%)	30 (25)	43 (44)	0.002

^1^Grade 3 or 4 of the SOFA score. SAPS II, simplified acute physiology score II; SOFA, sequential organ failure assessment.

Comparison of microbiological data did not demonstrate any significant difference except for significantly higher proportions of Candida species and lower proportions of staphylococci in those patients who underwent subsequent reoperation (Table 2). No significant change in the type or susceptibility profile of the species collected was detected over time during the study period (data not shown). The number of MDR bacteria and the proportions of MDR strains were not significantly different between the two groups of patients: 38 patients (31%) in the patients without reoperation versus 39 (40%) in the group with persistent sepsis (*P* = 0.18), Gram-negative MDR strains in 16 patients (13%) versus 20 (20%) (*P* = 0.19) and Gram-positive MDR strains in 29 patients (24%) versus 23 (23%), respectively (*P* = 0.95).

**Table 2:** **Microorganisms cultured from peritoneal fluid during initial septic surgery in patients without and with persistent peritonitis**

**Microorganisms**	**Without persistent peritonitis**	**Persistent peritonitis**	***P***
Aerobes, n (%)	292 (78)	209 (73)	0.17
Gram-positive bacteria, n (%)	141 (38)	94 (33)	0.21
Enterococci, n (%)	71 (19)	45 (16)	0.29
*E. faecalis, n (%)*	40 (11)	21 (7)	0.17
*E. faecium*, n (%)	16 (4)	12 (4)	1.0
Streptococci, n (%)	32 (9)	34 (12)	0.15
Staphylococci, n (%)	37 (10)	13 (5)	0.011
*Staphylococcus aureus*, n (%)	12 (3)	3 (1)	0.11
Coagulase-negative staphylococci, n (%)	25 (7)	10 (4)	0.08
Gram-negative bacteria, n (%)	151 (40)	115 (40)	0.98
*Enterobacteriaceae*, n (%)	122 (32)	95 (33)	0.83
*Escherichia coli*, n (%)	56 (15)	48 (17)	0.50
Enterobacter spp, n (%)	20 (5)	12 (4)	0.58
Klebsiella spp, n (%)	20 (5)	11 (4)	0.45
Morganella spp, n (%)	10 (3)	10 (4)	0.64
Proteus spp, n (%)	11 (3)	5 (2)	0.44
Non-fermenting Gram-negative bacilli, n (%)	24 (6)	12 (4)	0.23
*Pseudomonas aeruginosa*, n (%)	22 (6)	12 (4)	0.37
Anaerobes, n (%)	48 (13)	34 (12)	0.73
Bacteroides spp, n (%)	31 (8)	28 (10)	0.49
Fungi, n (%)	36 (10)	43 (15)	0.03
*Candida albicans*, n (%)	23 (6)	34 (12)	0.011
Candida non-albicans, n (%)	13 (3)	8 (3)	0.66
Total number of microorganisms, n	376	286	
Total number of MDR bacteria, n (%)	49 (13)	35 (14)	0.76

Results are expressed as number of isolates in each group.

**Figure 2:**
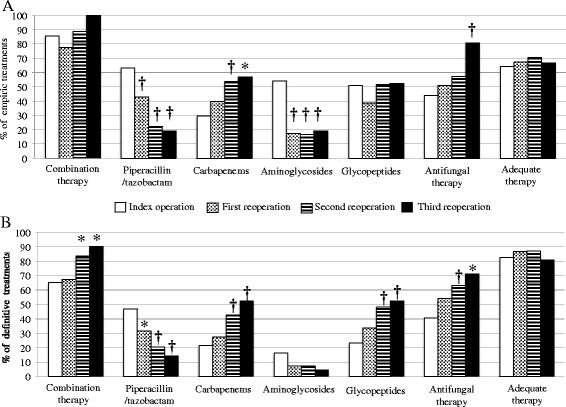
**Proportions of antibiotic therapies administered during initial surgery (S0) and at the time of reoperation (R1, R2 and R3), expressed as type of agent and adequacy of these regimens.** **(A)** Empirical antibiotic therapies. **(B)** Definitive antibiotic therapies. *P <0.05, ^†^ *P* <0.01 versus S0.

Adequacy of empirical antibiotic therapy was achieved in similar proportions in both groups (Table 1). Significantly increased proportions of broad-spectrum agents were used in the most severe cases of patients who subsequently underwent reoperation for persistent peritonitis (Figure 2). Among the 35 patients without persistent peritonitis who died, 25 (71%) had received adequate empirical antibiotic therapy versus 25 (61%) among the 41 patients with persistent peritonitis who died (*P* = 0.34). In addition, 15 patients (43%) who died without persistent peritonitis had a MDR strain, compared to 28 patients (68%) with persistent peritonitis (*P* = 0.025).

### Characteristics of patients with persistent sepsis at the time of additional reoperation

Following the index surgery, all 98 patients with persistent peritonitis underwent a first reoperation for persistent signs of abdominal sepsis, 54 of them required a second reoperation and 21 required a third reoperation (Figure 1). A total of 271 additional surgical procedures was analysed in these patients.

The clinical and surgical characteristics of the patients at the time of reoperation are shown in Table 3. The most frequent indication for reoperation was persistent signs of sepsis or septic shock. The times to reoperation remained stable throughout the study period. Inadequate source control related to anastomotic leakage or perforation was the most frequent cause of persistent abdominal sepsis. The aetiology of the failed source control at each reoperation, presented in Table 4, demonstrates the heterogeneity of the outcomes with no linear trends. Time to reoperation and clinical presentation at the time of reoperation were similar between the two groups.

**Table 3:** **Surgical and clinical characteristics at the time of the first, second and third repeat laparotomy for persistent peritonitis**

	**First repeat laparotomy**	**Second repeat laparotomy**	**Third repeat laparotomy**	***P***
	**n = 98**	**n = 54**	**n = 21**	
Time since previous surgery, days, mean ± SD	5 ± 5	6 ± 4	6 ± 5	0.32
Reasons for reoperation				
Sepsis, n (%)	23 (23)	17 (32)	3 (16)	0.27
Septic shock, n (%)	59 (60)	28 (55)	12 (63)	0.61
Other reasons, n (%)	16 (16)	9 (17)	6 (29)	0.40
Intraoperative diagnosis, n (%)				
Anastomotic leakage, n (%)	25 (26)	9 (17)	4 (21)	0.43
Perforation or ischaemia, n (%)	17 (17)	14 (26)	4 (21)	0.44
Purulent collection, n (%)	27 (28)	15 (28)	5 (26)	0.93
Other causes, n (%)	8 (7)	10 (18)	2 (10)	0.15
No cause, n (%)	21 (21)	6 (11)	6 (32)	0.15
Clinical characteristics at the time of reoperation				
Temperature, °C, mean ± SD	38.2 ± 1.2	38.1 ± 1.4	38.0 ± 1.5	0.53
WBCs, 10^9^/L, mean ± SD	19.5 ± 11.2	20.7 ± 10.6	21.6 ± 10.5	0.65
Creatinine (μmol/L)	159 ± 121	180 ± 129	202 ± 153	0.30
SOFA score, mean ± SD	7 ± 4	8 ± 4	9 ± 3	0.33
≥2 organ dysfunctions, n (%)	45 (46)	27 (50)	11 (52)	0.81
Haemodynamic failure^1^, n (%)	62 (63)	31 (57)	14 (67)	0.69
Respiratory failure^1^, n (%)	38 (39)	22 (41)	5 (24)	0.37
Renal failure^1^, n (%)	25 (26)	18 (33)	4 (21)	0.39

^1^Grade 3 or 4 of the SOFA score. WBCs, white blood cells; SOFA, sequential organ failure assessment.

**Table 4:** **Aetiology of failed source control in patients with persistent peritonitis, depending on the previous diagnosis**

**Index operation for postoperative peritonitis**	**First reoperation**	**Second reoperation**	**Third reoperation**
**n = 98**	**n = 98**	**n = 54**	**n = 21**
Bowel perforations n = 48	Bowel perforations n = 17	Bowel perforations n =14	Bowel perforations n =4
	12 bowel perforations at S0	3 bowel perforations at R1	1 purulent collection at R2
	5 suture leakages at S0	5 suture leakages at R1	2 suture leakages at R2
	2 purulent collections at S0	1 purulent collection at R1	1 other cause at R2
	4 other causes at S0	2 other causes at R1	
		3 no causes at R1	
Suture leakages n = 30	Suture leakages n = 25	Suture leakages n = 9	Suture leakages n = 4
	10 bowel perforations at S0	1 bowel perforation at R1	2 bowel perforations at R2
	11 suture leakages at S0	4 suture leakages at R1	2 suture leakages at R2
	5 purulent collections at S0	1 purulent collection at R1	
	4 other causes at S0	3 no causes at R1	
Purulent collections n = 20	Purulent collections n = 27	Purulent collections n = 15	Purulent collections n = 5
	13 bowel perforations at S0	2 bowel perforations at R1	2 bowel perforations at R2
	9 suture leakages at S0	4 suture leakages at R1	1 suture leakages at R2
	6 purulent collections at S0	4 purulent collections at R1	1 purulent collection at R2
	4 other causes at S0	2 other causes at R1	1 other cause at R2
		3 no causes at R1	
Other causes n = 19	Other causes n = 8	Other causes n = 10	Other causes n = 2
	3 suture leakages at S0	3 bowel perforations at R1	1 suture leakage at R2
	3 perforations at S0	3 suture leakages at R1	1 purulent collection at R2
	3 purulent collections at S0	2 purulent collections at R1	
	2 other causes at S0	1 other cause at R1	
	No causes n = 21	No causes n = 6	No causes n = 6
	13 bowel perforations at S0	3 purulent collections at R1	2 suture leakages at R2
	2 suture leakages at S0	2 suture leakages at R1	2 purulent collections at R2
	4 purulent collections at S0	1 no cause at R1	2 other causes at R2
	5 other causes at S0		

S0, index surgery; R1, first reoperation; R2, second reoperation; R3, third reoperation.

### Microbiological examinations in patients with persistent sepsis at the time of reoperation

Overall, 384 microorganisms (290 bacteria and 94 fungi) were cultured from surgical samples obtained during reoperation (Table 5). A significant and progressive decrease in the proportions of streptococci and anaerobes and increasing proportions of staphylococci, and NFGNB were observed from the index surgery to the third reoperation.

**Table 5:** **Microorganisms cultured from peritoneal fluid during index surgery and reoperation**

**Microorganisms**	**Index surgery**	**First reoperation**	**Second reoperation**	**Third reoperation**
Aerobes, n (%)	209 (73)	157 (71)	89 (75)	33 (75)
Gram-positive bacteria, n (%)	94 (33)	69 (31)	36 (31)	12 (27)
Enterococci, n (%)	45 (16)	38 (17)	18 (15)	4 (9)
*E. faecalis, n (%)*	21 (7)	11 (5)	8 (7)	0
*E. faecium*, n (%)	12 (4)	15 (7)	7 (6)	4 (9)
Streptococci, n (%)	34 (12)	7 (3)^†^	1 (1)^†^	0*
Staphylococci, n (%)	13 (5)	22 (10)*	16 (14)^†^	7 (16)^†^
*Staphylococcus aureus*, n (%)	3 (1)	1 (1)	1 (1)	1 (2)
Coagulase-negative staphylococci, n (%)	10 (4)	21 (9)	15 (13)	6 (14)
Gram-negative bacteria, n (%)	115 (40)	88 (40)	53 (45)	21 (48)
*Enterobacteriaceae*, n (%)	95 (33)	70 (32)	35 (30)	15 (34)
*Escherichia coli*, n (%)	48 (17)	35 (16)	18 (15)	4 (9)
Enterobacter spp, n (%)	12 (4)	10 (5)	7 (6)	4 (9)
Klebsiella spp, n (%)	11 (4)	9 (4)	4 (3)	2 (5)
Morganella spp, n (%)	10 (4)	6 (3)	2 (2)	1 (2)
Proteus spp, n (%)	5 (2)	2 (1)	2 (2)	1 (2)
Other *Enterobacteriaceae*, n (%)	9 (3)	8 (4)	2 (2)	3 (7)
Non-fermenting Gram-negative bacilli, n (%)	12 (4)	18 (8)	16 (14)^†^	5 (11)
*Pseudomonas aeruginosa*, n (%)	12 (4)	14 (6)	11 (9)	5 (11)
Acinetobacter spp, n (%)	0	4 (2)	3 (3)	0
Anaerobes, n (%)	34 (12)	8 (4)^†^	2 (2)^†^	1 (2)
Bacteroides spp, n (%)	28 (10)	5 (2)^†^	2 (2)^†^	1 (2)
Fungi, n (%)	43 (15)	57 (26)^†^	27 (23)	10 (23)
*Candida albicans*, n (%)	34 (12)	41 (18)*	22 (19)	8 (18)
Candida non-albicans, n (%)	8 (3)	14 (6)	4 (3)	2 (5)
Total number of microorganisms, n	286	222	118	44

*P <0.05, ^†^ *P* <0.01 versus index surgery.

The proportions of Candida isolates increased moderately but were similar in terms of numbers of patients harbouring fungi, as these organisms were cultured from 41 (41%) patients at the index surgery, 48 patients (49%) at the first reoperation, 26 patients (47%) at the second reoperation, and 9 patients (43%) at the third reoperation. The proportions of non-albicans strains remained stable at the various time-points (four *C. glabrata* at the time of the index operation, six *C. glabrata* and two *C. krusei* at the first reoperation, two *C. glabrata* and one *C. krusei* at the second reoperation). Among the 30 episodes of peritonitis from which staphylococci were cultured, staphylococci were associated with Candida species in 19 cases (63%). Similarly, among the 24 episodes in which NFGNB were isolated, NFGNB were associated with Candida species in 12 cases (50%).

### Emergence of microorganisms

**Figure 3:**
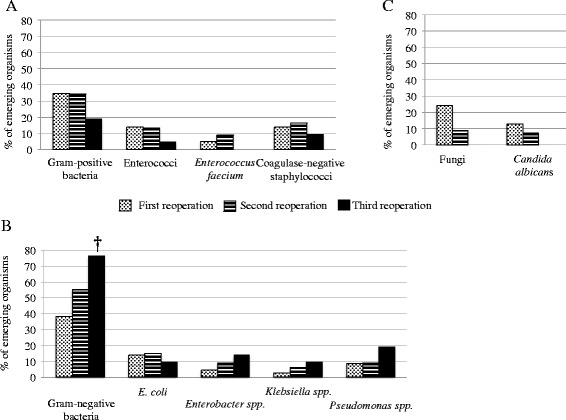
**Proportions of emerging microorganisms cultured from peritoneal samples expressed as proportions among their respective species at the time of reoperation (R1, R2 and R3).** **(A)** Gram-positive bacteria. **(B)** Gram-negative bacteria. **(C)** Fungi. *P <0.05, ^†^ *P* <0.01 versus index surgery.

A total of 203 emerging microorganisms were reported in 65 patients (66%)vat the first reoperation, 37 patients (69%)vat the second reoperation, and 13 patients (69%) at the third reoperation. In four patients, a change in susceptibility profile of previously identified and treated organisms can be hypothesized: one *E coli* strain at the first reoperation, and three strains at the second reoperation in three different patients *E. coli*, *M. morganii* and *P. aeruginosa*. However, no molecular investigation was performed to confirm these suspicions. The proportion of emerging microorganisms is presented in Figure 3. Although the emergence of new Gram-positive and Gram-negative bacteria was observed at each reoperation, the emergence of fungi was essentially reported at the first reoperation and only minimally at the second reoperation.

**Figure 4:**
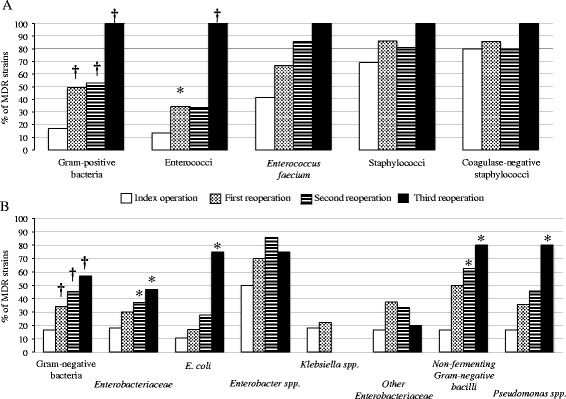
**Proportions of multidrug-resistant (MDR) bacteria cultured from peritoneal samples expressed as proportions among their respective species during initial surgery (S0) and at the time of reoperation (R1, R2 and R3).** **(A)** Gram-positive bacteria. **(B)** Gram-negative bacteria. *P <0.05, ^†^ *P* <0.01 versus S0.

Increasing proportions of MDR organisms were isolated from initial surgery to the third reoperation, involving Gram-positive and Gram-negative organisms (Figure 4). A total of 85 emerging MDR strains were cultured in 30 patients (30%) at the first reoperation, 24 patients (44%) at the second reoperation and 10 patients (48%) at the third reoperation. No vancomycin resistance was observed in any Gram-positive organism. Overall, among the 39 patients (41%) with MDR strains at the index surgery, the proportions of MDR strains increased to 48/98 (49%) at the first reoperation, 32/54 (54%) at the second reoperation (*P* = 0.037 versus S0) and 16/21 (76%) at the third reoperation (*P* = 0.003 versus S0).

### Adequacy of antibiotic therapy and duration of therapy

At the time of the index operation, no significant difference in terms of adequacy of antibiotic therapy was observed between patients with and without persistent peritonitis (Table 1). The type and adequacy of empirical and definitive antibiotic therapy from the index surgery to the third reoperation are shown in Figure 2. The microorganisms not targeted by empirical anti-infective therapy in patients with and without persistent peritonitis are presented in Table 6. No link was demonstrated between adequacy and reoperation. The average duration of antibiotic therapy for surviving patients was 11 ± 3 in the group without reoperation versus 13 ± 6 in the group with persistent peritonitis (*P* <0.01).

**Table 6:** **Microorganisms not targeted by empirical anti-infective therapy in patients without and with persistent peritonitis**

	**No persistent peritonitis**	**Persistent peritonitis**			
		**Index surgery**	**First reoperation**	**Second reoperation**	**Third reoperation**
Patients with inadequate therapy, n (%)	43 (35)	35 (36)	32 (33)	16 (30)	7 (33)
One organism not targeted, n (%)	29 (24)	10 (10)	26 (27)	11 (20)	6 (29)
Staphylococci, n (%)*	11 (3)	13 (5)	13 (6)	6 (5)	3 (7)
Methicillin-resistant coagulase-negative staphylocci, n (%)*	7 (2)	11 (4)	12 (5)	5 (4)	3 (7)
Enterococci, n (%)*	14 (4)	6 (2)	7 (3)	-	-
*Enterococcus faecium*, n (%)*	12 (3)	5 (2)	4 (2)		
Enterobacteriaceae, n (%)*	13 (3)	9 (3)	-	3 (3)	2 (5)
Non-fermenting Gram-negative bacilli, n (%)*	2 (1)	4 (1)	6 (3)	7 (6)	-
Candida species, n (%)*	18 (5)	13 (5)	12 (5)	5 (4)	2 (5)
*Candida albicans*, n (%)*	14 (4)	7 (2)	9 (4)	3 (3)	2 (5)

*Proportions expressed as number of isolates in each group versus total number of microorganisms.

### Risk factors for emergence of bacteria

Emergence of Gram-positive strains was more frequently observed in the case of monotherapy (22/46 (48%) versus 35/127 (28%) with combination therapy, *P* = 0.012), more specifically with the emergence of *Enterococcus avium* (5 (11%) versus 0 (0%), *P* = 0.0011). Emergence of Gram-positive strains was lower in the case of vancomycin therapy (5/49 (10%) versus 52/124 (42%) without vancomycin, *P* <0.0001), more specifically with the emergence of MRCNS (3 (6%) versus 26 (21%), *P* = 0.022). No specific risk factor was identified for emergence of Gram-negative bacteria. Emergence of *C. albicans* was more frequently observed in the case of monotherapy (10 (22%) versus 11 (9%), *P* = 0.032) and was lower in the case of vancomycin therapy (1/49 (2%) versus 20/124 (16%), *P* = 0.0089) and fluconazole therapy (5/82 (6%) versus 16/91 (18%), *P* = 0.033).

Risk factors for emergence of microorganisms were assessed in univariate analysis (Table 7). In multivariate analysis, a prolonged interval before reoperation was identified as a risk factor for emergence of microorganisms, but the presence of Candida appeared to play a protective role (Table 7).

**Table 7:** **Univariate and multivariate analyses of risk factors for emergence of microorganisms among the 173 reoperations in 98 patients with persistent peritonitis**

**Variable**	**Univariate analysis**						**Multivariate analysis**		
	**Missing data**	**Emerging organisms**	**No emerging organisms**	**Odds ratio**	**95% CI**	***P*** **-value**	**Odds ratio**	**95% CI**	***P*** **-value**
		**(n = 115)**	**(n = 58)**						
Time to reoperation, days	0	6 ± 5	4 ± 3	1.19	1.07, 1.35	0.001	1.19	1.06, 1.36	0.004
Initial infection involving *E. coli*	0	52 (45)	19 (33)	1.69	0.88, 3.32	0.14	-	-	-
Initial infection involving *Enterobacter* spp.	0	9 (8)	9 (16)	0.46	0.17, 1.25	0.18	-	-	-
Initial infection involving Candida	0	45 (39)	36 (62)	0.39	0.20, 0.75	0.0059	0.40	0.20, 0.77	0.007
Use of third-generation cephalosporins	0	7 (6)	9 (16)	0.35	0.12, 1.00	0.054	-	-	-
Use of glycopeptides	0	27 (23)	22 (38)	0.50	0.25, 0.99	0.051	-	-	-
Use of fluoroquinolones	0	13 (11)	1 (2)	7.26	1.39, 133.67	0.036	-	-	-
Inadequate anti-infective therapy	0	12 (10)	12 (21)	0.44	0.18, 1.07	0.10	-	-	-

Results are expressed as mean ± SD or total number (%). Time to reoperation (*P* = 0.0001 in univariate analysis), but not duration of therapy (*P* >0.2 in univariate analysis) was included in the model of risk factors for emergence of microorganisms. The variable, duration of therapy, was forced into the model but did not change the results. *C*-index: 0.71 (95% CI: 0.62, 0.79); Hosmer-Lemeshow test *P*-value: 0.19.

### Risk factors for emergence of MDR bacteria

Vancomycin therapy between index surgery and the first reoperation decreased the frequency of emergence of MDR strains (3/30 (10%) compared to 20/68 (29%) in patients without vancomycin (*P* = 0.041)). No specific risk factor was identified for emergence of MDR Gram-negative bacteria. Adequate antibiotic therapy did not change the frequency of emergence of MDR strains regardless of the period analysed. Risk factors for emergence of MDR organisms were assessed in univariate and multivariate analysis (Table 8).

**Table 8:** **Univariate and multivariate analyses of risk factors for emergence of MDR microorganisms among the 173 reoperations in 98 patients with persistent peritonitis**

**Variable**	**Univariate analysis**						**Multivariate analysis**		
	**Missing data**	**Emerging MDR strains (n = 63)**	**No emergence of MDR strains (n = 110)**	**Odds ratio**	**95% CI**	***P*** **-value**	**Odds ratio**	**95% CI**	***P*** **-value**
Type 1 diabetes mellitus	0	10 (16)	7 (6)	2.77	0.99, 7.08	0.061	-	-	-
SOFA score, per one point	0	8 ± 4	7 ± 3	1.07	0.97, 1.17	0.17	-	-	-
Time to reoperation, days	0	7 ± 6	4 ± 3	1.19	1.08, 1.32	<0.0001	1.19	1.08-1.33	0.0006
Polymicrobial initial infection	0	39 (62)	86 (78)	0.453	0.23, 0.89	0.034	-	-	-
Duration of anti-infective therapy, days	0	8 ± 5	6 ± 4	1.12	1.04, 1.21	0.0003	-	-	-
Use of third-generation cephalosporins	0	3 (5)	13 (12)	0.37	0.10, 1.36	0.17	-	-	-
Use of fluoroquinolones	0	9 (14)	5 (5)	3.5	1.12, 10.95	0.039	-	-	-

Results are expressed as mean ± SD or total number (%). An interaction between time to reoperation and duration of antibiotic therapy was identified (Pearson correlation coefficient between the two variables = 0.46 (95% CI 0.33, 0.57) (*P* <0.0001)). An interaction term was added in the multivariate analyses of risk factors for emergence of MDR microorganisms, which did not modify the results. *C*-index: 0.69 (95% CI: 0.61, 0.77); Hosmer-Lemeshow test *P*-value: 0.08. MDR, multidrug-resistant.

## Discussion

To the best of our knowledge, this study is the first to focus on susceptibility issues of the microorganisms isolated in patients with persistent peritonitis. Inadequate source control was identified as a leading cause of persistent peritonitis. Initial severity, the presence of Candida species and inadequate source control were the only risk factors for reoperation. A progressive shift in the cultured flora was observed with the number of reoperations, marked by extinction of susceptible streptococci and anaerobic strains, and the emergence of both Gram-negative and Gram-positive MDR strains and fungi. The interval between initial surgery and reoperation, and the presence of Candida appeared to play an important role as risk factors for emergence of microorganisms.

Our highly selected population and the retrospective study design could be considered to be a limitation, but a prospective multicentre study on this topic would appear to be unfeasible. Our findings on the patterns of empirical and definitive antibiotic usage, the characteristics of local microbial flora, or the hospital influence on the culture results have to be considered cautiously and cannot be directly generalised. The long study duration may have led to changes in antibiotic susceptibility patterns over time. However, such changes were not observed and environmental characteristics appeared to play only a minor role in our population. Our data did not distinguish between pathogenic strains and colonisation, which is particularly important for coagulase-negative staphylococci and yeasts. In clinical practice, prescribers usually target most, if not all, isolates. Another major limitation in the interpretation of our results is the lack of any pharmacokinetic monitoring in peritoneal fluid. We routinely monitored the plasma concentrations of antibiotics, but satisfactory plasma concentrations do not guarantee adequate local concentrations [23,24]. However, in the absence of data in the literature, we consider that our results provide an interesting overview of persistent abdominal sepsis in ICU patients, which could be helpful for physicians in the field.

Our failure to identify clear risk factors for persistent peritonitis is not surprising, as this study was not designed for this purpose. Several studies over the last decade have also failed to identify clear risk factors or surrogates for persistent peritonitis [4,25,26]*.* The time to reoperation varies considerably according to the literature, from 2 to 7 days [4,16,27], but some papers have reported delayed reoperations after up to 20 days [16]*.* In our cohort, times to reoperation were situated in the median range throughout the periods analysed. This variability suggests that time to reoperation is at least partially a physician-dependent variable, rather than a variable that reflects the course of the disease.

To our knowledge, the only study assessing the dynamics of peritoneal flora during persistent/recurrent abdominal sepsis used a different methodology [11]. Although our results are in agreement with those reported by these authors on the concept of changing peritoneal flora, we report different results in terms of Gram-negative strains and anaerobes. In addition, we provide a global analysis because of the high frequency of generalised peritonitis in most of our cases.

The present study presents the highest rate of MDR ever reported in the course of intra-abdominal infections [5,8,14,15,28-30]. The progressive extinction of highly susceptible strains such as streptococci and anaerobes [5,14,15,28,30] and the emergence of MDR strains [14,15,29] have been previously reported, but not to the extent observed here. Several risk factors promoting the emergence of MDR strains have been described, such as antibiotic therapy during the three months preceding surgery, prolonged hospital stay, previous and/or prolonged antibiotic therapy [12-15]. However, the timing of emergence of resistance strains has rarely been described. Seguin *et al*. suggested that a 5-day cut off in length of hospital stay or between the first operation and repeat laparotomy was the best predictive factor for the presence of MDR bacteria [13,14]. Our findings confirm this cut off and highlight the sustained and progressive increase in the proportions of MDR with the number of reoperations. This progression rate can be estimated to be about 15% between each reoperation, as illustrated by our sequential observations.

Among Gram-negative bacteria, MDR *Enterobacteriaceae,* mainly represented by *Enterobacter spp*. and *E coli*, were the predominant strains at the first reoperation. The importance of MDR strains of *E. coli* has been previously reported, but to a lesser extent, while the capacity of *Enterobacter spp*. to develop resistance is commonly observed [12,13,15,31]. The development of NFGNB, mainly *P. aeruginosa,* has been largely reported in the literature [12,13,15,31], although these organisms are usually observed in limited proportions [32]. Although their ability to develop resistance is well known, their progressive emergence from the first to the third reoperation to the detriment of *Enterobacteriaceae* is a noteworthy finding. These observations, well known in ventilator-associated pneumonia, are poorly documented in intra-abdominal infections.

The absence of MRSA among MDR Gram-positive cocci is another important finding compared to previous reports [15,31,33]. The decreased incidence of MRSA observed progressively over the last two decades in healthcare-associated infections in French institutions could explain this trend [34]. Coagulase-negative staphylococci, regularly mentioned in reports of tertiary peritonitis [2,5,7], were almost constantly observed in polymicrobial infections of the present study, suggesting that they only play a colonising role. In the absence of data concerning their pathogenicity, these strains were frequently taken into account in the choice of anti-infective therapy. The presence of *E. faecium* was largely expected, as these organisms are commonly reported in postoperative peritonitis, accounting for 30 to 50% of all Gram-positive cocci in previous reports [29,35], but the proportions resistant to amoxicillin and aminoglycosides were impressive.

Previous studies on tertiary peritonitis have reported the importance of yeasts [2,5,11]. The proportions of yeasts and the predominance of *C. albicans* remained stable from index surgery to the third reoperation. The great majority of patients treated by antifungal agents received fluconazole according to the recommendations available at the time. The use of fluconazole rather than a fungicidal agent might explain this high proportion of Candida isolates. We also observed a frequent association between Candida and staphylococci and NFGNB which may not be simply a coincidence, as mechanisms of synergy have been described experimentally between these organisms, [36,37]. The pathophysiology of persistent peritonitis remains poorly understood and some similarities with biofilms could be hypothesized, related to the presence of fibrin. The emerging concept of fungal prophylaxis in patients with recurrent abdominal perforation could be relevant in this setting [38,39].

Emerging organisms, especially MDR strains, have been commonly reported in the course of postoperative peritonitis [12-15,29,31] leading to the proposal of specific strategies targeting the largest number of organisms right from the empirical phase of treatment [12]. Interestingly, Gram-negative organisms were the predominant emerging strains. A progressive tapering in the emergence of Candida, and to a certain extent Gram-positive strains, was observed with no clear explanation. The potential protective role of Candida as a risk factor for emergence of microorganisms needs to be confirmed, as Candida peritonitis is also a factor for poor prognosis [28].

Finally, we did not identify any specific risk factors for emergence of MDR microorganisms compared to those previously reported [12-14]. Despite a cautious analysis of the relationships between antibiotic agents and emerging strains, we did not evidence a role of any specific class of antimicrobial agent. The only obvious finding was a decreased incidence of Gram-positive strains in the presence of vancomycin therapy. No recommendations can be proposed concerning the optimal antibiotic regimen on the basis of these findings. However, our observations led to a change in our approach to the use of anti-infective agents. Despite the absence of proof in the field of intra-abdominal infections, we now try to optimize the administration of antibiotic agents with increased dosing and prolonged or continuous infusion of beta-lactams [40]. In addition, we try to rapidly de-escalate our empirical therapy in order to preserve our antibiotic resources. Finally, we now use echinocandins as first-line therapy when fungal infection is suspected.

## Conclusions

Initial severity, the presence of Candida in surgical samples and inadequate source control are the major risk factors for persistent peritonitis during postoperative peritonitis. Our data suggest a progressive shift of peritoneal flora with the number of reoperations, comprising extinction of susceptible strains and emergence of both Gram-negative and Gram-positive MDR strains and fungi. Emergence of MDR bacteria is frequent and increases progressively with the number of reoperations. Surprisingly, no link was demonstrated between emergence of MDR strains and antibiotic regimens, while source control and its timing appear to be major determinants of the emergence of MDR strains. These observations could constitute an argument in favour of limited-spectrum empirical antibiotic therapy in the case of early reoperation in order to preserve the range of available anti-infective agents. A better understanding of intra-abdominal infections and their management requires additional tools to distinguish colonising organisms from infective pathogenic organisms in order to determine which organisms should be treated.

## Key messages

- Initial severity, presence of Candida and inadequate source control are the major risk factors leading to persistent abdominal sepsis during postoperative peritonitis
- Emergence of multidrug resistant microorganisms occurs rapidly during persistent abdominal sepsis
- The proportions of these multidrug-resistant microorganisms increase progressively with the number of reoperations for persistent abdominal sepsis

## Abbreviations

MDR: mutidrug resistant organisms
MRCNS: methicillin-resistant coagulase-negative staphylococci
MRSA: methicillin-resistant *Staphylococcus aureus*
OR: odds ratio
NFGNB: non-fermenting Gram-negative bacilli
SAPS II: simplified acute physiology score II
SOFA: sequential organ failure assessment
WBC: white blood cell
